# Microsurgery for intracranial arteriovenous malformation: Long-term outcomes in 445 patients

**DOI:** 10.1371/journal.pone.0174325

**Published:** 2017-03-21

**Authors:** Qingqing Ren, Min He, Yunhui Zeng, Zhiyong Liu, Hao Liu, Jianguo Xu

**Affiliations:** Department of Neurosurgery, West China Hospital, West China Medical School, Sichuan University, Chengdu, Sichuan, People’s Republic of China; Massachusetts General Hospital, UNITED STATES

## Abstract

**Background:**

The management of intracranial arteriovenous malformations(AVMs) poses challenges to the cerebrovascular specialists.

**Objective:**

To review the long-term outcomes of intracranial AVMs treated with microsurgical resections.

**Methods:**

We performed a retrospective review of 445 patients with intracranial AVMs treated in our hospital from January 1^st^, 2008 to December 31^st^, 2014. The extracted data included demographic characteristics, clinical presentations, Spetzler-Martin (SM) grades, Supplemented Spetzler-Martin (SM-Supp) Grades, treatment modalities, long-term outcomes, and obliteration rates. Outcome was assessed with a post-operative modified Rankin Scale (mRS) score at the last follow-up visit.

**Results:**

Of the 445 patients treated with microsurgery, 298 (67.0%) patients initially presented with hemorrhage. Based on the SM grading system, the patients were graded as follows: 83(18.6%) Grade I, 156(35.1%) Grade II, 132(29.7%) Grade III, 61(13.7%) Grade IV and 13(2.9%) Grade V. Overall, 344(77.3%) patients had a favorable outcome (mRS score of 0–2). The favorable outcome for Grade I and II were 92.8% and 85.9%, respectively, sharply reducing to 52.5% in patients with Grade IV and 15.4% in patients with Grade V AVMs. 388(87.2%) patients achieved complete obliteration of the AVMs. 63(14.2%) patients experienced recurrent hemorrhage, and the frequency of rehemorrhage was highest in Grade V patients (77.0%), dropping to 3.6% and 3.8% in patients with Grade I and II lesions, respectively. Permanent neurological deficits occurred in 66(14.8%) patients and death in 35(7.9%) patients. There was no difference of AUROC values between SM grading system and SM-supp grading system (0.726 and 0.734, respectively, p = .715).

**Conclusion:**

The Spetzler-Martin grading system is a simple and effective method to estimate the risk of surgery and to evaluate the prognosis. Microsurgical resection for AVMs depends on the SM grades, and the morbidity-mortality rate increases with an increasing SM grade.

## Introduction

Intracranial arteriovenous malformations(AVMs) are abnormalities of the intracranial vessels that compose tortuous arteries and veins, and lack an intervening capillary bed. AVMs are the most common type of intracranial vascular malformations, and the leading cause of nontraumatic intracerebral hemorrhages in young people less than 35 years old.[1] The incidence of AVMs in unselected population is about 1 per 100,000 per year.[2] AVMs account for 1% to 2% of all strokes, 3% of strokes in young adults, and 9% subarachnoid hemorrhages, and response for 4% of all primary intracerebral hemorrhages, but as much as one third in young adults.[3] The Most common clinical presentations of AVMs are intracranial hemorrhage and seizures, and typically presented before the age of 40 years old.[4] Focal neurologic deficits seem to occur in 1% to 40% of patients with intracranial AVMs, which can be transient, persistent or progressive.[5] The average yearly risk for first hemorrhage is between 2% and 3%,[6] while the rate of hemorrhage was 2.2% for unruptured AVMs and 4.5% for ruptured AVMs,[7] but the risk of recurrent hemorrhage may be as high as 34% in the first year.[8] Interventions including endovascular embolization techniques, stereotactic radiosurgery and microsurgery, have made great advances over the past decade, allowing effective multidisciplinary treatment of arteriovenous malformations.[9] Nevertheless, the morbidity is as high as 53% to 81%,[6, 10] and the mortality ranges from 10% to 30%,[11, 12] although some data suggest that the mortality rate may be lower.[13]

The management of intracranial arteriovenous malformations(AVMs) poses challenges to the cerebrovascular specialists.[14] Microsurgical resections remain the most effective and immediate treatment for AVMs.[15] Here we report a cohort of 450 patients with AVMs microsurgery resections in a single center over the past decade. We reviewed the long-term outcomes of these patients to evaluate the efficacy and safety of microsurgery resections for intracranial AVMs.

## Methods

### Patient population and data collection

With the approval of West China Hospital Institutional Ethical Review Board of Sichuan University, we undertook a retrospective review of our hospital database for all the patients diagnosed of intracranial AVMs who were treated with microsurgical resections during the period of January 1^st^, 2008 and December 31^st^, 2014. Written informed consent was obtained from each participating patient. Patients with any type of intracranial vascular malformations beyond AVMs as arteriovenous fistulas, vein of Galen malformations, or spinal vascular malformations were excluded from the analysis. A total of 445 patients were identified and patients baseline demographic, clinical, angiographic data were collected. The Spetzler-Martin (SM) grade was applied to all patients when admitted to the hospital to assess surgical risk,[16] and the Supplemented Spetzler-Martin (SM-Supp) grade was accessed retrospectively.[17] The duration of follow-up was noted, at the end of the follow-up (death time or on July 1^st^, 2016), a modified Rankin Scale (mRS) score was assigned and documented.[18]

### Surgical technique

When a patient was admitted to the hospital, the process of selecting the best therapy included microsurgery, endovascular therapy, and/or radiosurgery was made by a trained cerebrovascular neurosurgeon. The medical treatment decision was made based on both physician recommendations and patient preference. The desired goal of the treatment was permanently eliminating the AVM and preserving neurological functions. Assessment of AVM obliteration was determined by postoperative angiography.

Some patients with ruptured AVMs who were sent to the Emergency department received craniotomy and removal of hematoma prior to microsurgical resection. A portion of patients with planned staged treatment of the AVM underwent multimodality treatment with embolization or radiosurgery followed by microsurgery. Some patients received this procedure as a preoperative measure. However, a small group of patients was recommended for radiosurgery with/without endovascular treatment for residual AVMs after microsurgery.

### Statistical analysis

Statistical analysis was performed with Stata version 14.0 (StataCorp, College Station, Texas). Statistical analysis for categorical variables was performed with chi-square test or the Fisher exact test, and comparisons between mean values of continuous variables were performed with a t-test. Univariate analysis was used to test covariates predictive of postoperative complications. All multivariable models included adjustment for the duration of follow-up (log transformed time), which influenced final mRS assessments. The areas under the Receiver operating characteristic (ROC) curves were compared for accuracy in predicting change in mRS score. An area under the ROC curve of 1.0 indicates perfect discrimination, whereas an area of 0.5 indicates no discrimination. Generally, an area under the ROC curve of 0.70 or more is considered a clinically useful predictive model. Any p values of ≤ 0.05 were considered statistically significant.

## Results

### Baseline characteristics

A cohort consisted of 445 patients who were treated with intracranial AVMs microsurgery in our hospital from 1^st^ January 2008 to 31^st^ December 2014. Table 1 provides a summary of baseline characteristics of the cohort. Patients ranged in age from 1 year to 78 years, with an average age of 32 years (±16 years). There were 285(64.0%) males and 160(36.0%) females in the study group. 298(67.0%) patients initially presented with hemorrhage, 82(18.4%) patients complained of headache without hemorrhage, and 63(14.2%) presented with seizures. Some other presenting symptoms included visual disturbance, ataxia, hemiplegic paralysis, weakness, coma and altered mental status. 39(8.8%) patients were incidentally discovered without any symptoms. The AVM location was lobar in 320(71.9%), deep supratentorial in 67(15.1%), and cerebellar in 58(13.0%) of the cases.

**Table 1:** Baseline characteristics of all patients.

Parameters	Variable	Patients No. (%)
Sex	Male	285(64.0)
	Female	160(36.0)
Age	Mean: 32(±16.0) years	
	Range: 1–78 years	
Presentation	Hemorrhage	298(67.0)
	Seizure	63(14.2)
	Headache	82(18.4)
	Incidental	39(8.8)
Location	Lobar	320(71.9)
	Deep supratentorial	67(15.1)
	Cerebellar	58(13.0)

236(53.0%) of the AVMs nidus size were smaller than 3cm in diameter, 155(34.8%) were between 3-6cm, and 54(12.1%) were larger than 6cm in diameter, with an average of 3.3cm (±1.8cm). 186(41.8%) of the AVMs had deep venous drainage, and 40(9.0%) associated with an aneurysm either on a flow-related artery or an intracranial location. Eloquence of adjacent brain was considered in 206(46.3%) cases. Based on the SM grading system (Table 2) for AVMs, there were 83(18.6%) Grade I lesions, 156(35.1%) Grade II lesions, 132(29.7%) Grade III lesions, 61(13.7%) Grade IV lesions and 13(2.9%) Grade V lesions (Table 3).

**Table 2:** Points of variables according to Spetzler-Martin grading scale and supplementary Spetzler-Martin grading scale.

Variables	Spetzler-Martin Grading	Supplementary Spetzler-Martin Grading
Size, cm		
<3	1	1
3–6	2	2
>6	3	3
Venous drainage		
Superficial	0	0
Deep	1	1
Eloquence		
No	0	0
Yes	1	1
Age, y		
<20		1
20–40		2
>40		3
Bleeding		
Yes		0
No		1
Compactness		
Yes		0
No		1
Total	5	10

**Table 3:** Characteristics of arteriovenous malformations according to Spetzler-Martin grading scale and supplementary grading scale.

Variable	Patients No. (%)
AVM diameter	
<3cm	236(53.0)
3-6cm	155(34.8)
>6cm	54(12.1)
Venous drainage	
superficial	259(58.2)
deep	186(41.8)
Eloquence	
non-eloquent	239(53.7)
eloquent	206(46.3)
SM grade	
I	83(18.6)
II	156(35.1)
III	132(29.7)
IV	61(13.7)
V	13(2.9)
Age(Years)	
<20	107(24.1)
20–40	207(46.5)
>40	131(29.4)
Unruptured presentation	
Yes	147(33.0)
No	298(67.0)
Diffuse	
Yes	99(22.2)
No	346(77.8)
SM-Supp grade	
2	11(2.5)
3	34(7.6)
4	123(27.6)
5	130(29.2)
6	69(15.5)
7	52(11.7)
8	20(4.5)
9	4(0.9)
10	2(0.5)

A total of 207(46.5%) patients were aged between 20 and 40 years, with 107(24.1%) younger than 20 years and 131(29.4%) older than 40 years. 298(67.0%) patients had intracranial hemorrhage which indicated rupture of the AVMs, while 99(22.2%) were assessed as diffuse AVMs. According to the SM-Supp grading system (Table 2), 11(2.5%), 34(7.6%), 123(27.6%), 130(29.2%), 69(15.5%), 52(11.7%), 20(4.5%), 4(0.9%), 2(0.5%) of the cases were classified as score 2–10, respectively (Table 3).

### Clinical features and outcomes

Among all the 445 patients, 29(6.5%) underwent preoperative endovascular embolization of the AVMs, whereas 22(4.9%) had a single Gamma Knife radiation procedure prior to surgery, and Stereotactic radiosurgery (SRS) was performed on 5(1.1%) patients. Additionally, 36(8.1%) patients received craniotomy and removal of hematoma at the Emergency department, while 19(4.3%) had external ventricular drainage to decrease the intracranial pressure. Overall, 334(75.1%) patients underwent microsurgery resections only.

The follow-up time from completeness of surgery ranged from 1 day to 8.5 years, with a median of 4.6 years (±2.2 years). Postoperative cerebral angiogram including digital subtraction angiography (DSA), computed tomography angiography (CTA) and magnetic resonance angiography (MRA) was performed to assess the obliteration status. Of all the 445 patients, 388(87.2%) achieved complete obliteration of the AVMs, while 57(12.8%) had partial obliteration with residual arteriovenous shunting. Among the 57 patients with incomplete resections, 38(66.7%) underwent Gamma Knife radiation, 13(22.8%) underwent endovascular embolization, 2(3.5%) received repeat microsurgical resections, and 4(7.0%) had conservative management during the observational period.

The 1- and 5-year overall survival rates for patients with microsurgery were 95.3% and 92.1%, respectively. AVMs resulted in death in 35(7.9%) patients at the end of the follow-up time. 23(65.7%) of the 35 patients died of cerebral hernia caused by acute severe hemorrhage or re-hemorrhage, 7(20.0%) for infections (intracranial infection, pneumonia, fungi or bacteremia), 3(8.6%) for multiple organ failures, and 2(5.7%) gave up for financial conditions. Permanent neurological deficits occurred in 66(14.8%) patients, including 22 cases of hemiparesis, 6 of hemiplegia, 11 of aphasia, 5 of ablepsia, 13 of hydrocephalus, 3 of vegetative state and 6 local neurological dysfunctions. 11 of the 13 patients with hydrocephalus underwent postoperative ventriculoperitoneal shunt placement. Postoperative seizures occurred in 19(4.3%) patients and all under control after taking antiepileptic medication. 63(14.2%) patients experienced reoccurred hemorrhage.

**Fig 1:**
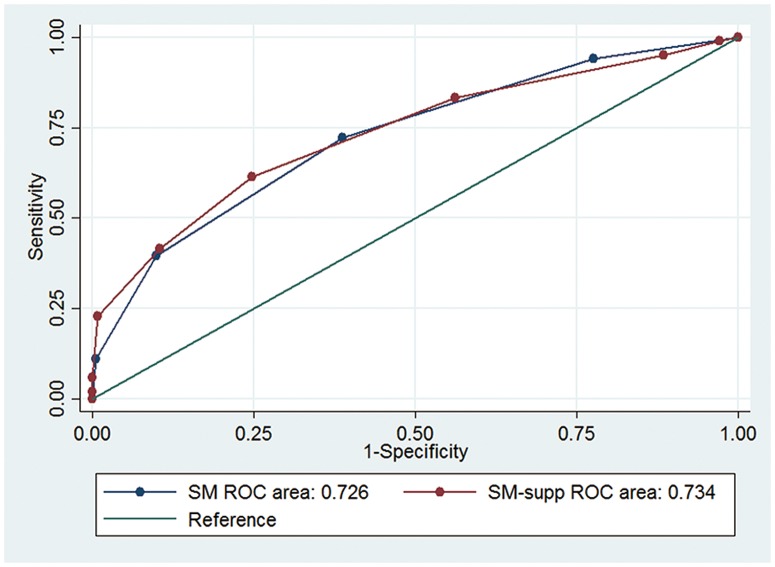
Graph showing receiver-operating characteristic analyses for the Spetzler-Martin grading system and the supplemented grading system. Graph showing receiver operating characteristic (ROC) analyses for the weighted point scores of the Spetzler-Martin grading system (green curve), and the supplementary grading system (red curve) (reference line shown in teal). The predictive accuracy of Spetzler-Martin score was similar to supplementary grade (ROC areas, 0.726 and 0.734, respectively, P = .715).

On presentation, 344(77.3%) patients had a favorable outcome (mRS score of 0–2).[19] The mRS of the entire cohort was as follows: 152(34.2%) patients had mRS of 0, 113(25.4%) had mRS of 1, 79(17.7%) had mRS of 2, 31(7.0%) had mRS of 3, 22(4.9%) had mRS of 4, 13(2.9%) had mRS of 5, and 35(7.9%) had mRS of 6. Univariable logistic regression analysis identified AVM size (P < .001), deep venous drainage (P < .001), age (P < .001) and diffuse nidus (P = .005) as significant predictors of poor outcome (mRS score >2) (Table 4). Whereas eloquence (P = .235) and unruptured presentation (P = .265) were not associated with poor outcome. In multivariate analysis, predictors of poor outcome (Table 5) were deep venous drainage (OR 2.46, 95% CI 1.40–4.35; p = .002), eloquence (OR 7.77, 95% CI 3.64–16.59; p < .001), age (OR 1.12, 95% CI 1.09–1.16; p < .001), unruptured AVMs (OR 2.51, 95% CI 1.27–4.97; p = .008) and diffuse nidus (OR 4.18, 95% CI 2.05–8.54; p < .001). The area under the ROC curve of SM grading system (0.726) was slightly lower than that of SM-supp grading system (0.734), but there was no significant difference between them (p = .715) (Fig 1).

**Table 4:** Univariable logistic regression analysis.

Variable	OR	95% CI		P value
Size	1.58	1.39	1.80	0.000
Drainage	2.68	1.69	4.21	0.000
Eloquence	1.31	0.84	2.04	0.235
Age	1.08	1.06	1.10	0.000
Unrupture	1.30	0.82	2.06	0.265
Diffuse	2.04	1.25	3.35	0.005

OR, odds ratio; CI, confidence interval

**Table 5:** Multivariable logistic regression analysis.

Variable	OR	95% CI		P value
Size	0.93	0.77	1.12	0.417
Drainage	2.47	1.41	4.33	0.002
Eloquence	7.34	3.48	15.50	0.000
Age	1.12	1.09	1.15	0.000
Unrupture	2.35	1.20	4.60	0.013
Diffuse	4.27	2.13	8.56	0.000

OR, odds ratio; CI, confidence interval

The SM grading system accurately correlated with long-term outcomes. The favorable outcome for Grade I and II were 92.8% and 85.9%, respectively, sharply reducing to 52.5% in patients with Grade IV and 15.4% in patients with Grade V AVMs (P = .004). The mortality rate of patients with Grade I AVMs was 0, increasing to 38.5% in patients with Grade V AVMs (P < .001). The frequency of rehemorrhage was highest in Grade V patients (77.0%), dropping to 3.6% and 3.8% in patients with Grade I and II lesions, respectively (P < .001).

## Discussion

### General characteristics

The incidence of intracranial AVMs is 1–1.4 cases per 100,000 person-years, and both sexes are affected equally.[2, 5, 20–28] The population of our group consisted of 285 males and 160 females, giving a 1.8:1 male preponderance, which is approximately similar to a Chinese population of 2086 patients with AVMs reported by Zhao et al in 2005[29]. The annual hemorrhage rate for unruptured and untreated brain AVMs is 2% to 4%, and the incidence of patients with brain AVMs presenting with intracranial hemorrhage is approximately 38% to 71%.[4, 7, 8, 20, 21, 27, 30–34] In our cohort, 67% of the patients presented with intracranial hemorrhage, and our result falls within this range. Based on our results, intracranial AVMs are typically diagnosed before the age of 40 years (70.6%), with hemorrhage (67.0%), headache (18.4%) and seizures (14.2%) as the presenting symptoms leading to the diagnosis.

### Microsurgery management and outcomes

AVMs were initially described by Luschka in 1854 and Virchow in 1863.[35, 36] Giordano performed the first open-surgery for an AVM and Péan performed the first surgically elimination of an AVM in 1889,[37] The treatment of intracranial AVMs including microsurgical resections, radiotherapy and endovascular embolization has made great advance over the past decades,[12, 38–44] but the management of intracranial AVMs remains controversial and challenging. The main treatment aim is to achieve complete angiographic obliteration of the AVMs, while preserving neurological functions.[9] Advantages of microsurgery are high rate of complete obliteration, and that it is the most time-tested and immediate treatment.[15, 39]

A systematic review and meta-analysis conducted by Beijnum and colleagues in 2011 reported that AVM obliteration was achieved in 96% (range 0% to 100%) of patients, with permanent neurological deficits or death occurring in a median 7.4% (range from 0% to 40%) of patients after microsurgery.[44] A meta-analysis comprising 2452 patients from 25 studies published between January 1990 and December 2000 showed a postoperative mortality of 3.3%, a permanent postoperative morbidity of 8.6%, varying from 1.5% to 18.7%, with postoperative angiography confirming a total excision of the AVM in 97% of the cases, varying from 91% to 100%.[45] Theofanis et al reported 264 patients treated with microsurgery and showed rates of permanent surgical morbidity and mortality of 1.9% and 2.7%, respectively, with an obliteration rate of 100%.[46]We presented a cohort comprising 445 patients treated with microsurgical resections in a large single center, and illustrated a permanent postoperative morbidity of 14.8% and a mortality of 7.9%. Even minor changes such as hydrocephalus or a small visual field deficit were assessed and rated as a deficit. All deaths were in patients who presented with hemorrhage and underwent urgent surgery, with a Glasgow coma scale of 7 or lower pre- or post-operation. Because ruptured brain AVMs presumably have a higher hemorrhage risk (4.5–34%) than unruptured ones (0.9–8%),[8] a high rate of hemorrhage presentation (67%) led to a high rate of rehemorrhage (14.2%), which both contributed to the disability and death, although presentation with AVM hemorrhage is an underappreciated predictor of outcome after microsurgical resection,[47]

A Randomised trial of Unruptured Brain Arteriovenous malformations (ARUBA) is a multicentre, non-blinded, randomized trial aimed to evaluate the risk of patients with an unruptured brain AVM treated with medical management alone compared to medical management with interventional therapy.[48] The ARUBA trial was started on April 4, 2007, and was halted on April 15, 2013 after an interim analysis showed that medical management alone is superior to medical management with interventional therapy (microsurgery, embolization, or radiosurgery) for the prevention of death or stroke in patients with unruptured brain AVMs followed up for 33 months. However, the results of ARUBA have provoked severe debates over concerns for weaknesses in study design, treatment modality, follow-up length, and external validity.[49–57] Of 223 patients with interim analysis when ARUBA was halted, 114 assigned to interventional therapy and 109 to medical management, only 18 patients were treated with neurosurgery. It’s criticized that only 5 patients treated surgically alone when 76 patients had an SM score of 1 or 2.[49] There were 147 patients with unruptured brain AVMs in our study, and 89 (60.5%) patients with SM Grade I or II received microsurgical resections and 78 (87.6%) developed good outcome with an mRS score of 0–2. However, a pragmatic randomized controlled trial started on March 25, 2014 is currently recruiting participants.[58] Treatment of Brain Arteriovenous Malformations Study (TOBAS) is a study including two randomized controlled trials and a registry to test the risk of death or debilitating stroke for unruptured AVMs treated with medical management compared to interventional therapy and to test if endovascular treatment can improve the safety and efficacy of surgery or radiation therapy.[59]

### Spetzler-Martin grading and supplemented Spetzler-Martin grading system

In 1986, Spetzler and Martin introduced a classification system based on lesion size, location, and venous drainage to evaluate surgical risk for intracranial AVMs (Table 2).[60] Spetzler and Ponce introduced a simplified and consolidated 3-tier classification for AVMs: class A, including the Grade I and II Spetzler-Martin AVMs; class B, including the Grade III Spetzler-Martin AVMs; and class C, including the Grade IV and V Spetzler-Martin AVMs.[61] Many other grading scales have been proposed,[62–72] however, Spetzler-Martin five-points scale is the most popular and widely used grading system.[73] Microsurgical resection of Grade I-II AVMs offers an immediate cure with very low procedural morbidity, while Grade IV–V AVMs are associated with unacceptably high morbidity and mortality rates, and the morbidity-mortality rate increases with an increasing Spetzler-Martin's grade.[45] Experience suggested that many important factors were interrelated. In 2010, Lawton et al introduced a supplementary grading system including additional variables such as patient age, hemorrhagic presentation, nidal diffuseness, and deep perforating arterial supply.[17] The supplementary grading system is a better predictor of neurologic outcomes after AVM surgery than the Spetzler-Martin grading system, and an SM-Supp grade of 6 is a cutoff or boundary for decision of microsurgery.[74]

In our series, the AUROC value for the SM-Supp system was a little better than for the SM system alone (AUROC = 0.734 vs 0.726; P = .715; Fig 1), which demonstrates that there is no difference in predictive accuracy between SM system and SM-Supp system. Female sex is another factor affects the outcomes of the microsurgery in the multivariate analysis, nevertheless, it is not included in SM-supp grading system. The SM-Supp model performed equally well in predicting risk of microsurgery, although Kim et al reported that the SM-Supp model was certified performing better than current prediction models.[74, 75]

## Limitations

Limitations include that the study is a retrospective review in a single-center, and the recall bias. The surgeries were operated by different neurosurgeons.

## Conclusion

The Spetzler-Martin grading system is a simple and effective method to estimate the risk of surgery and to evaluate the prognosis. Microsurgical resection for AVMs depends on the SM grades, and the morbidity-mortality rate increases with an increasing SM grade.
